# Multiparametric ultrasound for non-invasive evaluation of kidney graft function

**DOI:** 10.1007/s40477-025-00989-x

**Published:** 2025-04-04

**Authors:** Maria Irene Bellini, Sergio Angeletti, Daniele Fresilli, Mattia Di Segni, Gian Marco Lo Conte, Raponi Flavia, Manuela Garofalo, Renzo Pretagostini, Corrado De Vito, Patrizia Pacini, Vito D’Andrea, Angelo Barbato, Francesco M. Drudi, Marcello Caratozzolo, Vito Cantisani

**Affiliations:** 1 Department of Surgery, Sapienza University of Rome, 00161 Rome, Italy; 2 Department of Radiological, Oncological, and Pathological Sciences, Sapienza University of Rome, Viale Regina Elena 324, 00161 Rome, Italy; 3 Department of Radiology, Ospedale S. Scolastica, Via S. Pasquale, 03043 Cassino, Frosinone Italy; 4 Department of General and Specialty Surgery, Sapienza University of Rome, Viale Regina Elena 324, 00161 Rome, Italy; 5 Department of Public Health and Infectious Diseases, Sapienza University of Rome, 00185 Rome, Italy; 6 ASL Rieti, via del Terminillo 42, Rieti, Italy

**Keywords:** Renal transplant, Acute rejection, CEUS, Renal vascularization, Acute rejection

## Abstract

**Background:**

Renal transplant (RT) remains the optimal treatment for end-stage renal disease and early complications might be detected in the postoperative period to improve long-term outcomes. To this regard, contrast enhanced ultrasound (CEUS) could be utilized to evaluate RT functional recovery and potentially detect acute rejection (AR) and/or renal ischemia signs.

**Materials and methods:**

Observational study of 107 consecutive patients waitlisted for RT. Participants underwent conventional ultrasound (CUS) and color-doppler-ultrasound (CDUS) to evaluate resistive index of segmental and interlobar arteries and quantitative CEUS techniques recording the following perfusion parameters: peak intensity (PI-c), rising time (RT-c), area under (AUC-c) the time intensity curve (TIC), time to peak (TTP-c) and mean transit time (MTT-c).

**Results:**

CEUS parameters sensibility and specificity to predict AR in the early post-operative period resulted in: 90% and 69% for PI-c, 95% and 64% for RT-c, 85% and 65% for AUC-c. The overall diagnostic performance of these three CEUS parameters in comparison to the same in CUS and CDUS resulted in a sensitivity and specificity of 95% and 49%, versus 85% and 46%, respectively, therefore CEUS examination with the analysis of PI-c, RT-c and AUC-c values increases the diagnostic sensitivity in predicting AR by approximately 15–20% compared to CDUS and by 30–40% compared to CUS.

**Conclusion:**

CEUS could be routinely included in RT follow-up, as it shows to be a non-invasive helpful diagnostic tool for early detection of renal graft complications, selecting patients eventually in need of further confirmation.

## Introduction

Renal transplant (RT) represents the treatment of choice for selected end-stage renal disease, improving patients’ survival and quality of life [1].

Some grade of graft dysfunction occurs in 3–40% RT recipients, mainly in relation to the quality of the transplanted organ [2]. Delayed graft function is a potential threat to long-term graft survival, often because of an underlying rejection not early recognized and treated [3].

Diagnosis of acute rejection (AR) is histology. Unfortunately, renal biopsy is an invasive procedure, which may lead to complications such as hematoma, excessive blood loss, and even graft loss [4]. Furthermore, it is limited to local tissue sample which cannot represent the pathological condition of the entire organ.

In this clinical setting, diagnostic imaging plays an important role in early diagnosis of graft dysfunction by evaluating the entire RT in a non- or minimally invasive modality, and obtaining useful information for treatment and follow-up [5].

Color-Doppler-ultrasound (CDUS) is a low-cost and highly accessible method for graft vascular assessment, via the measurement of segmental and interlobar artery resistive indexes (RIs). Importantly, CDUS could be performed safely at bedside and without risks for the patient. Yet, CDUS does not directly analyze the cortical and medullary microcirculation, where instead the pathological alterations are located.

The contrast medium administration during the US examination (contrast enhanced ultrasound; CEUS) allows the visualization of tissue perfusion at the capillary level and quantifies renal blood flow, using kinetic parameters from time-intensity curves (TIC) [6].

In contrast, computed tomography (CT) and magnetic resonance imaging (MRI) can evaluate the cortical and medullary vascularization only in a phasic way, without a continuous and real-time evaluation. They are also associated with higher costs, low accessibility, and some relative contraindications, i.e. allergic reactions and/or adverse events following gadolinium-related nephrogenic systemic fibrosis and contrast agent-induced nephropathy.

The aim of this study is to compare CEUS characteristics with clinical and laboratory features, in order to assess post-transplant outcomes, focusing on early diagnosis of acute rejection (AR) and renal ischemia.

## Patients and methods

The study was conducted in accordance with the Declaration of Helsinki and the relevant guidelines and regulations. The clinical question was structured using the PICO methodology [7] (Table 1).

**Table 1:** PICO methodology used

P	Patient	Patients with end-stage renal disease undergoing renal transplant
I	Intervention	Early post-operative evaluation with multiparametric ultrasound (CUS, CDUS and CEUS)
C	Comparison	Clinical follow-up for patients with normal graft function Histology for patients with delayed functional recovery
O	Outcome	Should CEUS be included in the diagnostic follow up for renal transplant, in the early diagnosis of acute rejection and in the selection of patients requiring biopsy?

Observational study of consecutive patients waitlisted. Before initiating the study, all participants provided a written informed consent to be included in the study, according to the following criteria of inclusion: (1) age ≥ 18 years; (2) being transplanted in our hospital; (3) undergoing CUS and CEUS examination within seven days after RT and exclusion: (1) CUS or CEUS examination data not available or incomplete; (2) allograft kidney affected by transplant renal artery stenosis (TRAS) and/or cortical ischemia at CEUS; (3)no signs of rejection in histology.

All included patients underwent clinical, laboratory and ultrasound evaluation with CUS, CDUS and CEUS techniques.

Among the included patients, those who had at least one of the following conditions underwent renal biopsy:

1. Required supportive dialysis within the first 7 days after RT;
2. Serum creatinine (Cr) still > 400 μmol/L on postoperative day 7;
3. Postoperative urine output < 1200 mL/day, or decreased Cr < 10% for 3 consecutive days within seven days of surgery.

## Clinical and laboratory data collection

Demographic and laboratory characteristics from recruited participants were collected including age, gender, body mass index (BMI) and concomitant diseases. Laboratory parameters examined to evaluate renal function recovery consisted of: Cr, Delta Cr (pre- and 48 h after-RT), blood urea nitrogen (BUN), estimated glomerular filtration rate (eGFR) and urinary output.

## Multiparametric ultrasound

Each patient's CUS, CDUS and CEUS examinations were performed within seven days of RT by an experienced physician with more than 20 years of ultrasound and CEUS experience using “SAMSUNG, RS80 and 85 Prestige^®^” with a curved array transducer from 2 to 5 MHz also equipped with its own software, developed by “Samsung”, capable of processing the Wash-in and Wash-out Time Intensity Curves.

### CUS and CDUS

Initially, CUS and CDUS sonography were performed. The length and width of the graft were measured through visualization in grayscale, and RI values (peak systolic velocity and peak diastolic velocity) of the segmental artery and interlobar artery were calculated using pulse-wave Doppler in a long axis with a relative usual mechanical index (MI). The interlobar artery RI value was measured at least three times (at the upper, middle, and lower poles) and the average RI value was then recorded. The vascular anastomosis was also evaluated by Doppler evaluating the renal artery both at the renal hilum and at the ostium. The Doppler spectrum was considered optimal when at least three similar consecutive waveforms were visualized.

### CEUS

CEUS examination was performed using the same 2–5 MHz curved array transducer with an intravenous bolus injection of 1.5–2 mL ultrasound contrast SonoVue^®^ (Bracco, Milan, Italy) through a > 20 Gauge catheter followed by an additional 5–10 mL saline to wash the catheter. The long axis section of the renal graft was chosen to show the renal hilus and the maximum area of the graft for CEUS examination. CEUS was performed at a low MI technique (0.07). Patients were requested to take shallow and steady breaths during the examination.

The image acquisition process was initiated immediately after injecting the contrast agent; 120 s of real-time images were continuously captured and stored in digital imaging and communications in medicine (DICOM) format and used for qualitative evaluation.

Two 5 mm^2^ regions of interest (ROIs) were placed over the cortex and medulla respectively, and the TIC were generated by CEUS software and stored for quantitative analysis of each ROI [7].

The following perfusion parameters were recorded:

- Peak Intensity (PI), the maximum intensity of the TIC;
- Rising Time (RT), the time required to increase from 10 to 90% of PI after perfusion;
- Area Under the TIC (AUC), proportionate to the total volume of blood in the ROI;
- Time to Peak (TTP), the time from injection to the peak of the TIC;
- Mean Transit Time (MTT), the time for PI to drop by half.

## Statistical analysis

Statistical analysis was performed using STATA 17.0 software (StataCorp LLC, College Station, TX, USA). The diagnostic values of each parametric tool were assessed by calculating the area under the receiver operating characteristic curves (AUROCs). The optimal cutoff value was selected according to the best Youden index, and the diagnostic accuracy was calculated ((sensitivity, specificity, PPV, NPV, area under the curve (AUC)).

Diagnostic differences between different imaging methods were evaluated through Bonferroni test, and a *p*-value (*p*) < 0.05 was considered statistically significant.

## Results

**Fig. 1:**
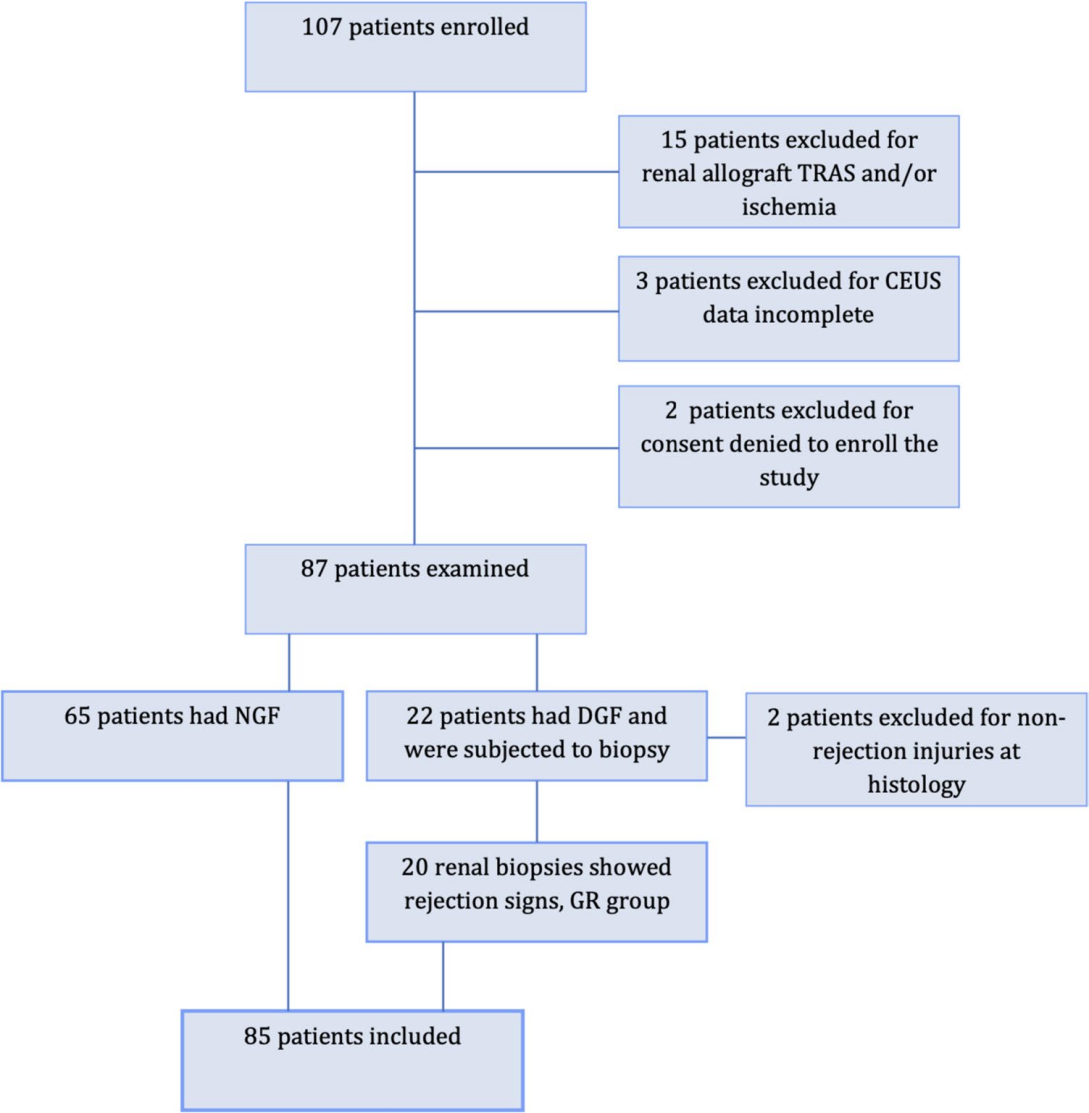
Flow chart of patient enrollment

The original population comprehended 107 consecutive patients waitlisted to undergo RT in the study period (Fig. 1). Twenty patients did not enter the study: 15 for TRAS and/or cortical ischemia; 3 for incomplete CEUS data and 2 because of denied consent to be enrolled. Eighty-five patients were finally included in the analysis (50 males and 35 females, with a mean age of 50.78 ± 13.56 years).

Patients excluded for TRAS and/or ischemia (5 with TRAS, 6 with ischemia and 4 with both TRAS and parenchymal ischemia) were analyzed as a subgroup to evaluate the CUS, CDUS and CEUS performances. CT with contrast-enhanced was done to confirm TRAS and diagnosis of cortical ischemia.

Sixty-five of 87 patients (75%) had a postoperative normal functional recovery (normal graft function, NGF group) and 22 patients (25%) showed delayed graft function, (DGF group). The DGF group underwent renal biopsy.

20 of 22 specimens showed signs of histological rejection (graft rejection, GR group) and only 2 shew non-rejection parenchymal damage, therefore these were excluded from the final analysis, as limited in sample.

## Clinical and laboratory parameters

There were no significant differences between the NGF group and DGF group in age, sex, BMI and concomitant diseases at baseline. The average levels of Delta Cr (pre- and 48 h post-RT) and urinary output between the two groups were 96.8 ± 88 μmol/L vs − 246.4 μmol/L ± 120 μmol/L and 41 ± 22 cc/h vs 157 ± 68 cc/h, respectively. The comparison between the Cr before RT and after 48 h, was almost stable in DGF (equal to approximately + 96.8 μmol/L) with an average Cr at discharge of 212.1 μmol/L. In the NGF group, Cr decreased approximately − 246.4 μmol/L with an average creatinine level of approximately 128.5 μmol/L.

## CUS results

No significant difference was observed in the renal allograft size between the DGF group and the NGF group (all *p* > 0.05). Cortical edema (CEd) was present in 11 of 20 patients with AR and in 16 patients of NGF, showing a sensitivity and specificity of 55% and 75% respectively.

## CDUS results

Doppler ultrasound demonstrated the best cut-off of segmental RI according to Youden was 0.72, determining a sensitivity of 60% and specificity of 65% to predict the renal allograft rejection in the early post-operative period.

The best cut-off of interlobar RI according to Youden was 0.73, determining a sensitivity of 75% and specificity of 52% to predict AR in the early post-operative period.

The interlobar RI value of the renal allograft was significantly higher in the GR group than in the NGF group (*p* = 0.007) with values of 0.73 ± 0.15 vs 0.66 ± 0.09, (*p* = 0.007), respectively.

On the contrary, the segmental RI value was slightly higher but not statistically significant (0.72 ± 0.21 versus 0.62 ± 0.11, *p* = 0.051).

## Quantitative CEUS Results

CEUS quantitative parameters are obtained from TIC on CEUS and their best cut offs were calculated according to the Youden test to maximize their diagnostic performance and the best are PI-c, RT-c, AUC-c (*p* < 0.05).

The best cut-off of cortical PI (PI-c) according to Youden was 21.75 dB, determining a sensitivity of 90% and specificity of 69% to predict AR in the early post-operative period.

Cortical PI was significantly lower in the GR group than in the NGF group (*p* = 0.003) with values of 15.12 dB ± 7.1 dB versus 19.5 dB ± 3.4 dB (*p* = 0.003).

The best cut-off of cortical RT (RT-c) according to Youden was 7.3 s, determining a sensitivity of 95% and specificity of 64% to predict AR in the early post-operative period.

Cortical RT is significantly shorter in the GR group than in the NGF group (*p* = 0.001) with values of 7.1 s ± 2.1 s versus 7.6 s ± 2.3 s, (*p* = 0.001).

The best cut-off of cortical AUC (AUC-c) according to Youden is 781.30 dB·seconds determining a sensitivity of 85% and specificity of 65% to predict AR in the early post-operative period.

Cortical AUC is significantly lower in the GR group than in the NGF group (*p* = 0.002) with values of 863.8 dB·seconds ± 401.8 dB·seconds versus 999. dB·seconds ± 265.4 dB·seconds, (*p* = 0 0.002).

There was no significant CEUS parameter difference of other CEUS parameters between DGF and NGF groups (all *p* > 0.05). Complete results are listed in Table 2.

**Table 2:** Results of conventional US, CDUS and quantitative CEUS

	AUC ROC Area	95% CI AUC	Sensitivity (%)	Specificity (%)	PPV %	NPV %
CUS						
Cortical Edema (CEd)	0.651	0.528–0.775	55	75.38	40.7	84.5
CDUS						
RI Segmental A	0.623	0.498–0.747	60	64.62	34.3	84
RI Interlobary A	0.636	0.521–0.751	75	52.31	32.6	87.2
CEd + RI Interlobary	0.656	0.555–0.757	85	46.2	32.7	90.9
CEUS						
PI-c	0.796	0.708–0.884	90	69.23	47.4	95.7
RT-c	0.790	0.713–0.867	95	63.8	44.2	97.6
AUC-c	0.748	0.648–0.847	85	64.62	42.5	93.3
TTP-c	0.569	0.443–0.695	60	53.85	28.6	81.4
MTT-c	0.625	0.502–0.747	65	60	33.3	84.8
PI-m	0.619	0.499–0.739	70	53.8	31.8	85.4
RT-m	0.690	0.576–0.804	75	63.1	38.5	89.1
AUC-m	0.638	0.529–0.747	80	47.69	32	88.6
TTP-m	0.551	0.424–0.679	55	55.38	27.5	80
MTT-m	0.642	0.522–0.761	70	58.46	34.1	86.4
PIc + RTc + AUCc	0.721	0.642–0.799	95	49.23	36.5	97

We also analyzed the overall diagnostic performance of the best three CEUS parameters (PI-c, RT-c and AUC-c) by comparing with the CUS and CDUS, and results a sensitivity of 95% and specificity of 49% for CEUS parameters (PIc + RTc + AUCc) and a sensitivity of 85% and specificity of 46% for CUS + CDUS.

**Fig. 2:**
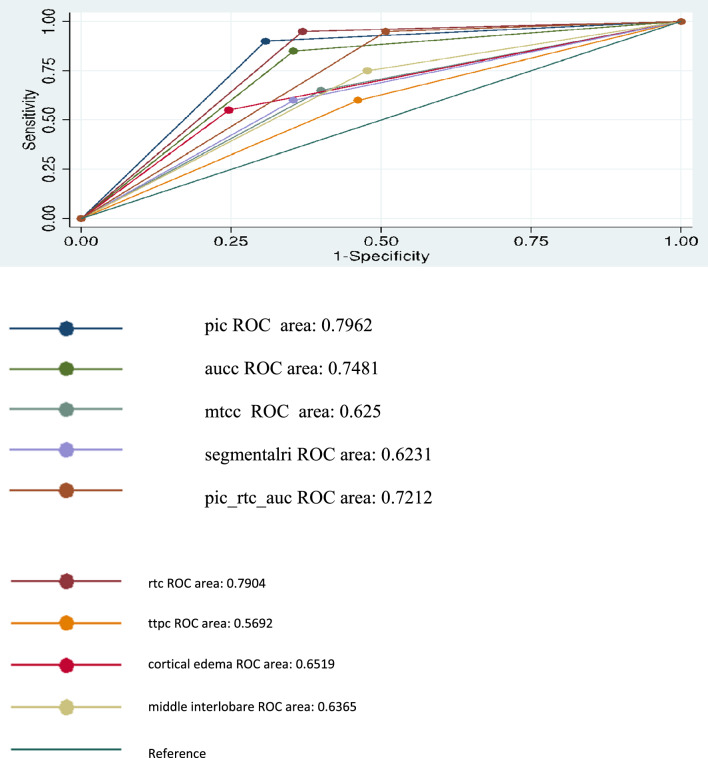
ROC curves comparison between conventional US, CDUS and quantitative CEUS

Comparing ROC curves of each method (Fig. 2, Table 3) we observed that the PIc represents the best parameter with an AUROC of 0.796 even better than the sum of the PIc + RTc + AUCc (0.721) without statistically significant difference. The statistically significant result is achieved by comparing PIc with MTTc (AUROC: 0.6250), TTPc (0.5692) and CDUS (0.6231 for RI segmental arteries and 0.6365 for RI interlobar arteries) (*p* < 0.05).

**Table 3:** Comparison quantitative CEUS, CDUS and CUS versus PIc for RA diagnosis by AUROC and Bonferroni test

	ROC Area	Std. Err	Chi2	df	Pr > chi2	Bonferroni Pr > chi2
pic (standard)	0.7962	0.0449	0.0229	1	0.8796	1.0000
rtc	0.7904	0.0392	1.4108	1	0.2349	1.0000
aucc	0.7481	0.0507	14.1769	1	0.0002	0.0013
ttpc	0.5692	0.0643	6.7333	1	0.0095	0.0757
mttc	0.6250	0.0627	5.4457	1	0.0196	0.1569
cortical edema	0.6519	0.0631	9.0893	1	0.0026	0.0206
segmental ri	0.6231	0.0636	9.0096	1	0.0027	0.0215
Interlobar ri	0.6365	0.0587	0.0229	1	0.8796	1.0000
pic_rtc_auc	0.7212	0.0400	4.5000	1	0.0339	0.2712

Comparing the ROC curves of the three best CEUS parameters alone (PIc, RTc and AUCc) with CUS associated with CDUS (Table 4), they are better with a statistically significant difference (*p* < 0.05).

**Table 4:** Comparison quantitative CEUS versus CUS + CDUS for RA diagnosis by AUROC and Bonferroni test

	ROC Area	Std. Err	Chi^2^	df	Pr > chi^2^	Bonferroni Pr > chi^2^
ce_int-a (standard)	0.6558	0.0515				
pic	0.7962	0.0449	11.7379	1	0.0006	0.0018
rtc	0.7904	0.0392	8.1843	1	0.0042	0.0127
aucc	0.7481	0.0507	5.9612	1	0.0146	0.0439

## TRAS and cortical ischemia subgroup

**Fig. 3:**
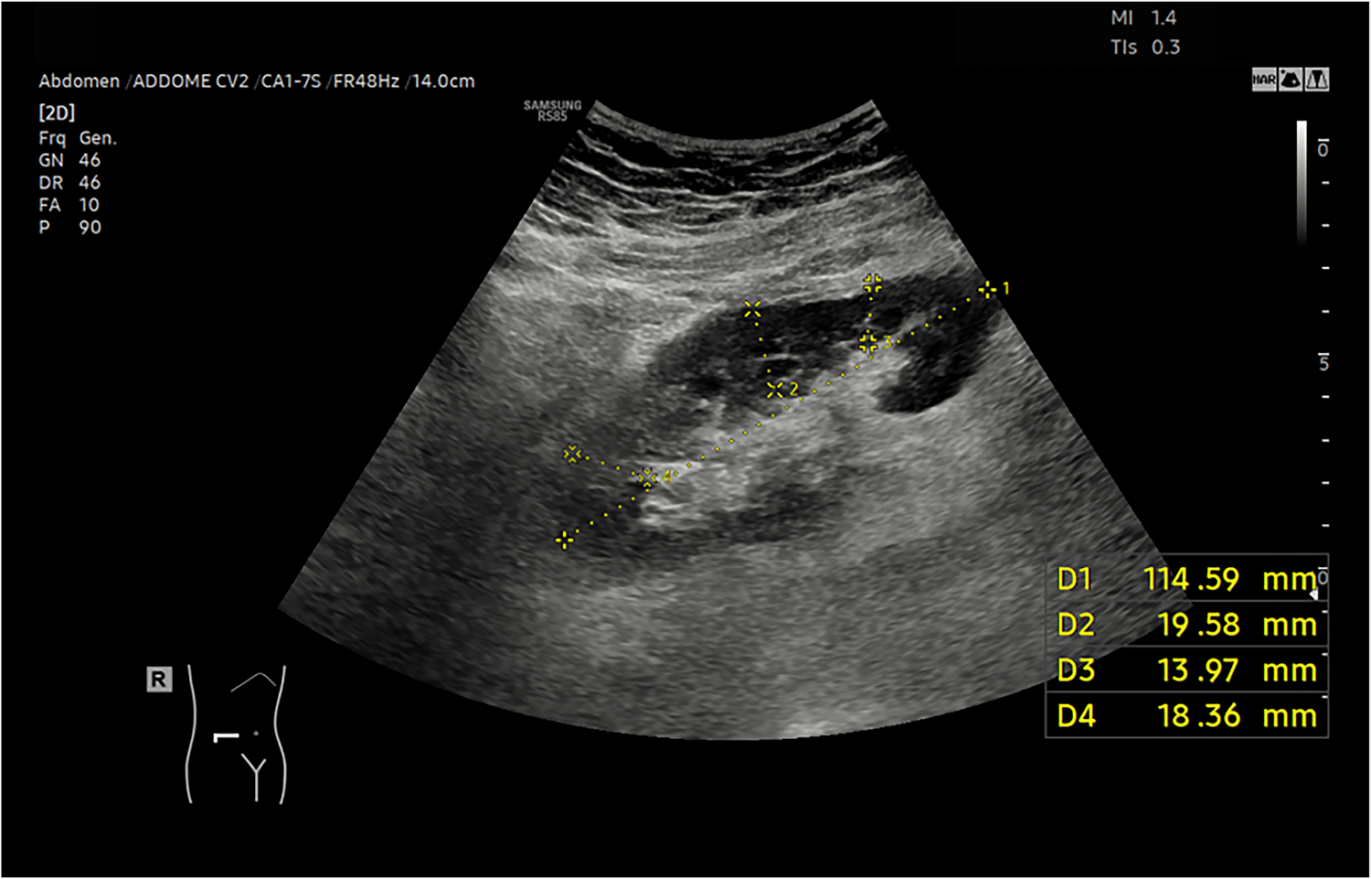
The CUS reveals slight thinning of the renal cortex in a Patient whit renal ischemia after renal transplantation

**Fig. 4:**
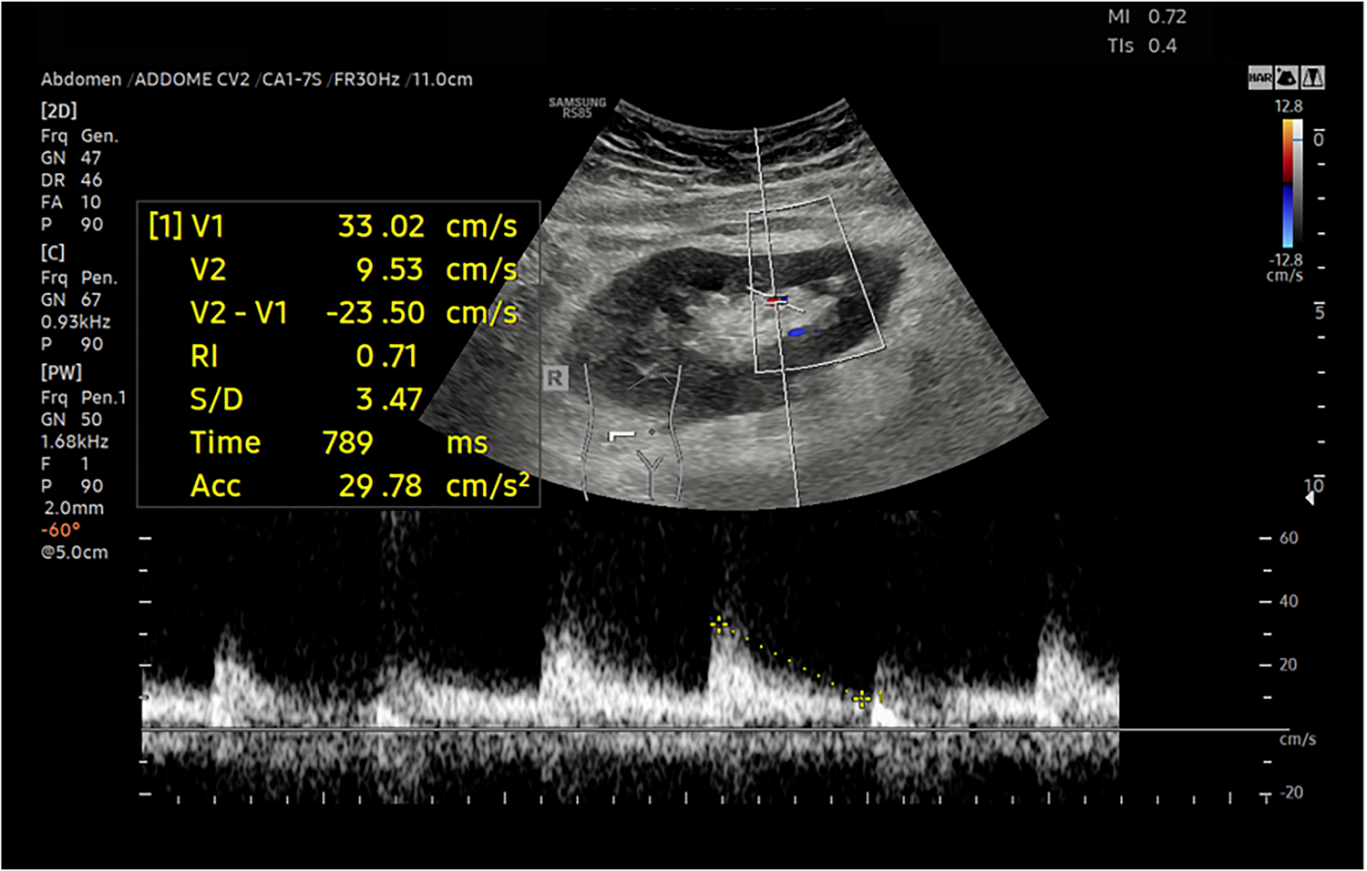
The Color-Doppler shows normal RI at the level of the segmental arteries in a Patient whit renal ischemia after renal transplantation

**Fig. 5:**
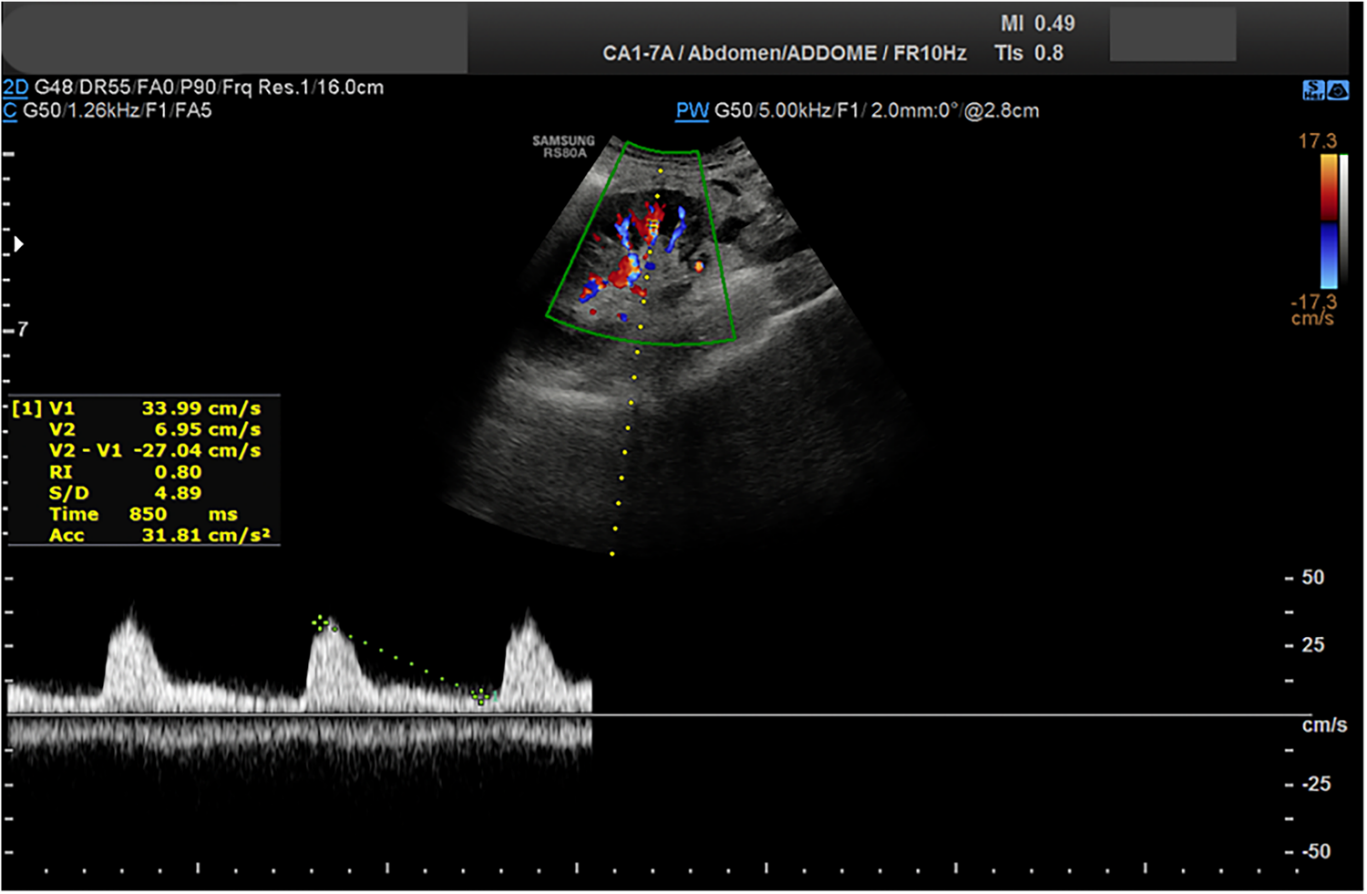
The Color-Doppler shows normal RI at the level of the segmental arteries in a Patient whit renal ischemia after renal transplantation

**Fig. 6:**
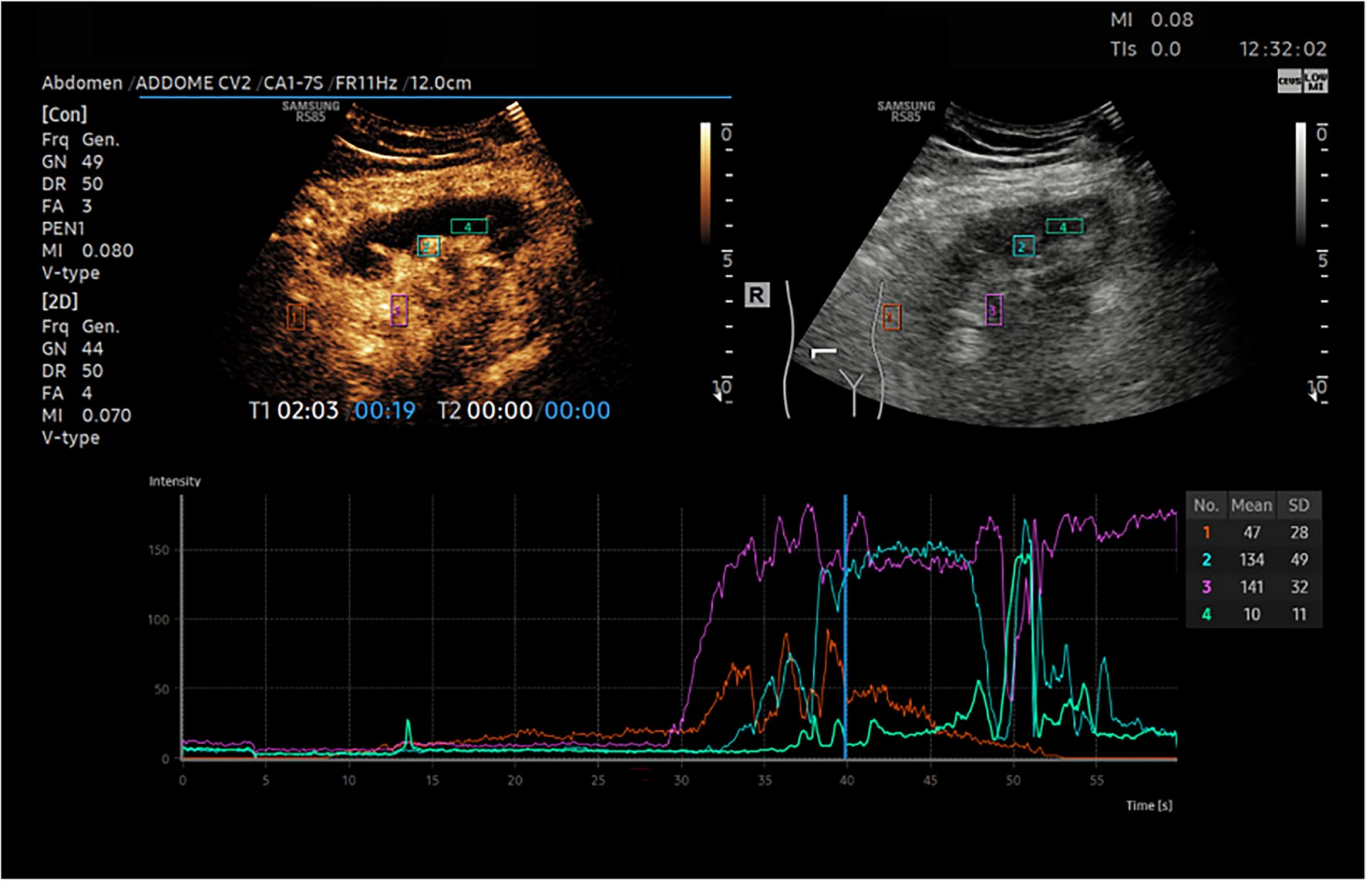
Qualitative and quantitative CEUS shows no enhancement at the lower pole of transplanted kidney in a Patient whit renal ischemia after renal transplantation

**Fig. 7:**
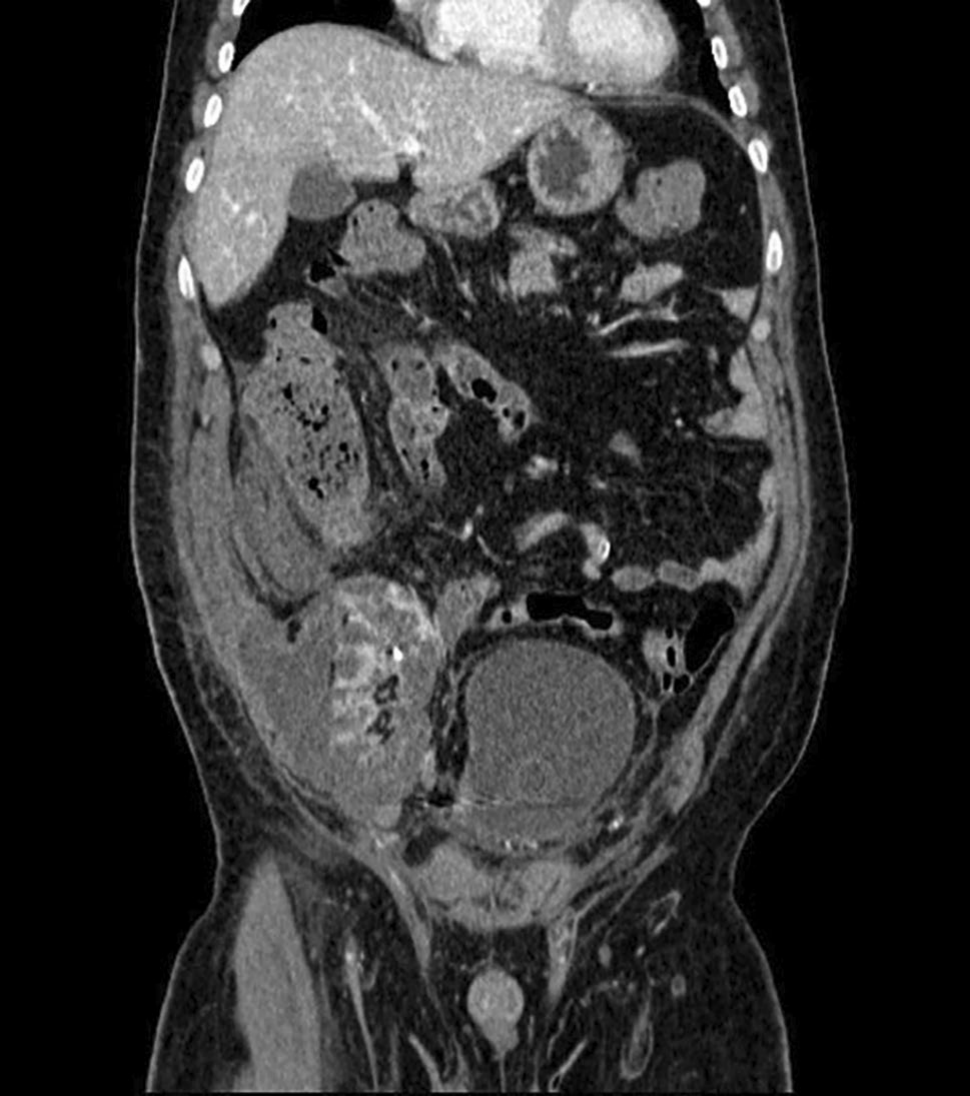
CT angiography confirms renal ischemia of lower pole of transplanted kidney

Fifteen patients with TRAS and/or cortical ischemia in the early post-operative period of the RT were not included in the final analysis, in accordance with the exclusion criteria used. In particular five patients have isolated TRAS, four TRAS associated with parenchymal ischemia and six isolated parenchymal ischemia. All cases are confirmed by CT angiography. CEUS identified all parenchymal ischemia [8], that instead were poorly assessable or not assessed by CUS and CDUS. CUS identified 6 of 10 cortical ischemia (60%) and CDUS 8 of 10 (80%). Nine patients with TRAS were present. CEUS correctly identified 8 cases (89%) while CDUS 7 (78%) and CUS only 4 (44%). Both CEUS and CDUS are results better than CUS in evaluating TRAS favoring CEUS in one case. (Figs. 3, 4, 5, 6, 7).

## Discussion

Ways to detect subclinical rejection are essential to monitor graft function in patients following KT, and protocol biopsies for surveillance are an invasive and potential harmful option, that need to be left only for patients where a significant AR concern is flagged. Yet, to monitor the immune response only by serial measurements of kidney function, namely serum creatinine, has low power to early predict signs of rejection and injury, therefore in the recent years, alternative non-invasive ways, such as the use of biomarkers, have been tested. Since, these last have no univocal interpretation for prospective patient management [9], sensitive and specific indicators of GR are very much advocated.

Ultrasound of renal allografts is a standard clinical method applied to manage RT patients noninvasively, but it has serious limitations related to intra-interoperation variability and no specific finding. Renal blood flow, vascular resistance and elastic compliance of bigger intrarenal vessels can be assessed by CDUS with RI measurement [10].

Unfortunately, RIs are influenced by both intra- and extra-renal parameters including vascular compliance, age, atherosclerosis, renal artery stenosis, pulse pressure, heart rate and rhythm [11], with some studies affirming their usefulness [12, 13] and others, in contrast, not sustaining it [14].

In expert hands, CDUS can be a valid tool for the diagnosis and follow-up assessment of all vascular complications following RT, given the possibility to assess increased speed in TRAS, distal spectral lengthening, and increased arterial acceleration time of intra-parenchymal arterial vessels [5].

Different MRI-based approaches have been tested for non-invasive kidney graft function evaluation [15] and although MRI is considered a promising technique in nephrology, it has not yet been established in the routine follow-up of RT [16].

Differently, CT is commonly available and generates X-ray-based images with high spatial resolution of the body. A CT scan can assess renal perfusion, but needs the administration of an intravenous contrast agent. However, as CT-based techniques rely on radiation and IV-contrast agents themselves can cause harm, CT has not yet been established in the clinical management of AR of renal allografts either [17].

On the other hand, several allografts’ pathologies are related to vascular remodeling and microvascular inflammation in the small parenchymal arteries and arterioles that are another reason why RIs measured on larger vessels (interlobar, segmental and arcuate arteries) are not the best parameter to detect microcirculatory alterations [18].

CEUS is a promising technique to assess renal microvascularization and it is playing an increasing role in the assessment of RT complications such as AR. Basically, CEUS improves standard ultrasonography enhancing the blood echogenicity through intravenous administration of microbubble-based contrast agents without potential nephrotoxic effects of iodinated contrast agents used in CT. Some authors, like Mueller-Peltzer et al., demonstrated CEUS to have a sensitivity of 85.7% and a specificity of 100% in diagnosing AR in comparison to transplant biopsy [19].

CEUS also assesses perirenal fluid and all the parenchymal abnormalities related to AR, including acute tubular necrosis, vascular complications, and parenchymal tumors. Furthermore, CEUS allows the detection of other vascular abnormalities accounting for 5–10% of all post-transplant complications, namely anastomosis stenosis, thrombosis of the renal vein and anatomical variants, such as a hypertrophied column of Bertin [20].

CEUS gives a real-time assessment of contrast enhancement (CE) and the continuous acquisition allows the representation of the cortico-medullary phase in all exams, regardless of the patient's hemodynamic status and without the need for bolus tracking. Continuous data acquisition allows the processing of TIC to evaluate tissue CE as a function of time, from which quantitative perfusion indices and renal blood flow kinetic parameters can be extracted and potentially useful for diagnosis and follow-up.

As demonstrated by our study, the best quantitative parameters (*p* value < 0.05) for AR diagnosis are AUC, PI e RT.

AUC represents the relative blood volume of the tissue in a certain ROI, which is correlated with the volume of contrast agents, blood flow velocity, and time of perfusion.

PI represents the microbubbles concentration in a certain ROI, which reflects the volume of perfusion.

RT represents the time required to increase from 10 to 90% of PI after perfusion in a certain ROI which reflects the velocity of perfusion.

Cortical AUC, PI and RT are reduced in AR groups, which indicates that less contrast microbubbles entered in the cortical microvascular bed of renal allograft in a specific unit of time.

In fact, the cortical CE is decreased with parenchymal rejection and its reduction is at the beginning for tubulitis, interstitial inflammation, glomerulitis, peritubular capillaritis, and arteritis [21] and later on following glomerulosclerosis, tubular atrophy and interstitial fibrosis, altogether resulting in cortical hypoperfusion [22].

The overall diagnostic performance of the three best CEUS parameters (PI-c, RT-c and AUC-c) both alone and combined are better than CUS and CDUS both alone and combined. In our study, the sum of Pic, RTc and AUCc shows an overall sensitivity of 95% increasing the sensitivity of CUS + interlobar CDUS (85%) by approximately 20%. This is in accordance with previous study reporting that quantitative CEUS parameters could help in the evaluation of the severity of pathologic changes in chronic kidney disease [23].

Due to its characteristics of safety, easy execution, repeatability and low cost, CEUS can be the first-line examination to evaluate post-RT complications, selecting patients for timely intervention or regular follow-up.

Although CEUS is proving to be a valid aid in the post-contrast evaluation of the RT, there are some limitations that should be taken into account:

- There is no uniform consensus on how to draw the ROI (circular, square or irregular using manually trackball-guided cursor technique);
- There is no uniform consensus on where to draw the ROI (in the cortex, in the medulla, between cortex and medulla, in the intrarenal arteries, regardless of internal kidney structure);
- CEUS requires sufficient skill in the examination technique, and therefore it could be poorly used in the short term period;
- CEUS is less panoramic compared to CT and MRI.

In our experience, we used an average of multiple ROIs of 5 mm2 in the cortex and medulla. In fact, as also suggested by Friedl et al. [24], the method of ROI 5 mm2 offers a standardized form and a sufficient, feasible size, enabling TIC analysis with low intraoperator and interoperator variance. However, studies comparing different ROI placements, sizes, and shapes are needed to standardize the TIC analysis protocol.

In the TRAS subgroup, CEUS identified almost all stenoses (89%), performing slightly better than CDUS which identified 78% and better than CUS (44%). In the subgroup of cortical ischemia, CEUS identified all parenchymal ischemia (100%) and was better than CUS (60%) and CDUS (80%).

To date, there is no evidence showing significant superiority of CEUS over CDUS in the diagnosis of TRAS. Color Doppler US can demonstrate increased speed in TRAS, distal spectral lengthening, and increased arterial acceleration time of intra-parenchymal arterial vessels.

The cut-off for pathologic TRAS peak systolic speed varies between 200 and 300 cm/s according to different authors. The lower value suffers from low specificity and can be responsible for an excessive number of superfluous investigations. Controversy still remains as to the best RI cut-off.

CT angiography can be used to assess the exact location and degree of stenosis for possible subsequent interventional digital subtraction angiography. In this context, MRI angiography is a powerful alternative for detecting TRAS, although this imaging modality is less accessible, and may overestimate the degree of stenosis.

In conclusion, CEUS is an effective imaging technique to evaluate post-RT disease without radiation exposure and nephrotoxic effects, and it could become a strong diagnostic method following ALARA (As Low As Reasonably Achievable) criteria. In particular, the evaluation by CEUS examination with the analysis of PI-c, RT-c and AUC-c values increases the diagnostic sensitivity in predicting AR by approximately 15–20% compared to the CDUS by evaluating the RIs and by 30–40% compared to CUS.

We therefore suggest including CEUS examination in the RT follow up from the very early period, as it could detect AR signs.

## Data Availability

Data may be available on reasonable request.

## Abbreviations

c: Cortical
m: Medullary
AR: Acute rejection
AUC: Area under the TIC
BMI: Body mass index
BUN: Blood urea nitrogen
CDUS: Color-doppler-ultrasound
CE: Contrast enhancement
CEd: Cortical edema
CEUS: Contrast enhanced ultrasound
Cr: Creatinine
CT: Computed tomography
CUS: Conventional ultrasound
DICOM: Digital imaging and communications in medicine
DGF: Delayed graft function
eGFR: Estimated glomerular filtration rate
GR: Graft rejection
MI: Mechanical index
MRI: Magnetic resonance imaging
MTT: Mean transit time
NGF: Normal graft function
NPV: Negative predictive value
OR: Odds ratio
p: P-value
PI: Peak intensity
PPV: Positive predictive value
RI: Resistive index
ROI: Region of interest
RT: Renal transplant
RT: Rising time
TIC: Time intensity curve
TRAS: Transplant renal artery stenosis
TTP: Time to peak
US: Ultrasound

## References

[1] Bellini MI, Courtney AE, McCaughan JA (2020) Living donor kidney transplantation improves graft and recipient survival in patients with multiple kidney transplants. J Clin Med 9(7):2118 32635614 10.3390/jcm9072118 PMC7408952

[2] Bellini MI, Tortorici F, Amabile MI, D’Andrea V (2021) Assessing kidney graft viability and its cells metabolism during machine perfusion. Int J Mol Sci 22(3):1121 33498732 10.3390/ijms22031121 PMC7865666

[3] Hilbrands L, Budde K, Bellini MI, Diekmann F, Furian L, Grinyó J et al (2022) Allograft function as endpoint for clinical trials in kidney transplantation. Transpl Int. 10.3389/ti.2022.10139 35669976 10.3389/ti.2022.10139 PMC9163811

[4] Trajceska L, Severova-Andreevska G, Dzekova-Vidimliski P, Nikolov I, Selim G, Spasovski G et al (2019) Complications and risks of percutaneous renal biopsy. Open Access Maced J Med Sci 7(6):992–995 30976347 10.3889/oamjms.2019.226 PMC6454172

[5] David E, Del Gaudio G, Drudi FM, Dolcetti V, Pacini P, Granata A et al (2022) Contrast enhanced ultrasound compared with MRI and CT in the evaluation of post-renal transplant complications. Tomography 8(4):1704–1715 35894008 10.3390/tomography8040143 PMC9326620

[6] Sidhu PS, Cantisani V, Dietrich CF, Gilja OH, Saftoiu A, Bartels E et al (2018) The EFSUMB Guidelines and Recommendations for the Clinical Practice of Contrast-Enhanced Ultrasound (CEUS) in non-hepatic applications: update 2017 (Long Version). Ultraschall Med 39(2):e2–e44 29510439 10.1055/a-0586-1107

[7] Richardson WS, Wilson MC, Nishikawa J, Hayward RS (1995) The well-built clinical question: a key to evidence-based decisions. ACP J Club 123(3):A12–A13 7582737 10.7326/ACPJC-1995-123-3-A12

[8] Alasfar S, Kodali L, Schinstock CA (2023) Current therapies in kidney transplant rejection. J Clin Med 12(15):4927 37568328 10.3390/jcm12154927 PMC10419508

[9] Park S, Sellares J, Tinel C, Anglicheau D, Bestard O, Friedewald JJ (2024) European Society of Organ Transplantation consensus statement on testing for non-invasive diagnosis of kidney allograft rejection. Transpl Int. 10.3389/ti.2023.12115 38239762 10.3389/ti.2023.12115 PMC10794444

[10] Meier M, Fricke L, Eikenbusch K, Smith E, Kramer J, Lehnert H et al (2017) The serial duplex index improves differential diagnosis of acute renal transplant dysfunction. J Ultrasound Med 36(8):1607–1615 28370148 10.7863/ultra.16.07032

[11] Viazzi F, Leoncini G Fau - Derchi LE, Derchi Le Fau - Pontremoli R, Pontremoli R. Ultrasound Doppler renal resistive index: a useful tool for the management of the hypertensive patient. (1473–5598 (Electronic)) 10.1097/HJH.0b013e328365b29c PMC3868026 24172238

[12] Bellini MI, Charalampidis S, Herbert PE, Bonatsos V, Crane J, Muthusamy A et al (2019) Cold pulsatile machine perfusion versus static cold storage in kidney transplantation: a single centre experience. Biomed Res Int 2019:7435248 30792996 10.1155/2019/7435248 PMC6354149

[13] Preuss S, Rother C, Renders L, Wagenpfeil S, Büttner-Herold M, Slotta-Huspenina J, et al. Sonography of the renal allograft: Correlation between Doppler sonographic resistance index (RI) and histopathology. (1875–8622 (Electronic)) 10.3233/CH-189306 30562894

[14] Naesens M, Heylen L Fau - Lerut E, Lerut E Fau - Claes K, Claes K Fau - De Wever L, De Wever L Fau - Claus F, Claus F Fau - Oyen R, et al. Intrarenal resistive index after renal transplantation. (1533–4406 (Electronic))

[15] Köhnke R, Kentrup D, Schütte-Nütgen K, Schäfers M, Schnöckel U, Hoerr V, et al. Update on imaging-based diagnosis of acute renal allograft rejection. (2160–8407 (Print)) PMC6526365 31139495

[16] van Eijs MJM, van Zuilen AD, de Boer A, Froeling M, Nguyen TQ, Joles JA, et al. Innovative Perspective: Gadolinium-Free Magnetic Resonance Imaging in Long-Term Follow-Up after Kidney Transplantation. (1664–042X (Print)) 10.3389/fphys.2017.00296 PMC5432553 28559850

[17] Thölking G, Schuette-Nuetgen K, Kentrup D, Pawelski H, Reuter S. Imaging-based diagnosis of acute renal allograft rejection. (2220–3230 (Print)) 10.5500/wjt.v6.i1.174 PMC4801793 27011915

[18] Harvey Cj Fau - Sidhu PS, Sidhu Ps Fau - Bachmann Nielsen M, Bachmann Nielsen M. Contrast-enhanced ultrasound in renal transplants: applications and future directions. (1438–8782 (Electronic)) 10.1055/s-0033-1350138 23929378

[19] Mueller-Peltzer K, Negrão de Figueiredo G, Fischereder M, Habicht A, Rübenthaler J, Clevert DA. Vascular rejection in renal transplant: Diagnostic value of contrast-enhanced ultrasound (CEUS) compared to biopsy. (1875–8622 (Electronic)) 10.3233/CH-189115 29630540

[20] Ghazanfar A, Tavakoli A Fau - Augustine T, Augustine T Fau - Pararajasingam R, Pararajasingam R Fau - Riad H, Riad H Fau - Chalmers N, Chalmers N. Management of transplant renal artery stenosis and its impact on long-term allograft survival: a single-centre experience. (1460–2385 (Electronic)) 10.1093/ndt/gfq393 20601365

[21] Jeong HA-O. Diagnosis of renal transplant rejection: Banff classification and beyond. (2211–9132 (Print)) 10.23876/j.krcp.20.003 PMC7105630 32164120

[22] Yang CA-O, Wu S, Yang P, Shang G, Qi R, Xu M, et al. Prediction of renal allograft chronic rejection using a model based on contrast-enhanced ultrasonography. (1549–8719 (Electronic)) 10.1111/micc.12544 PMC6767498 30887637

[23] Yang WQ, Mou S, Xu L, Li FH, Li HL. Prediction of tubulointerstitial injury in chronic kidney disease using a non-invasive model: combination of renal sonography and laboratory biomarkers. (1879–291X (Electronic)) 10.1016/j.ultrasmedbio.2018.01.019 29503020

[24] Friedl S, Jung EM, Bergler T, Tews HC, Banas MC, Banas B, et al. Factors influencing the time-intensity curve analysis of contrast-enhanced ultrasound in kidney transplanted patients: Toward a standardized contrast-enhanced ultrasound examination. (2296–858X (Print)) 10.3389/fmed.2022.928567 PMC9452686 36091698

