# Correction: Multiparametric ultrasound for non‑invasive evaluation of kidney graft function

**DOI:** 10.1007/s40477-025-01054-3

**Published:** 2025-07-21

**Authors:** Maria Irene Bellini, Sergio Angeletti, Daniele Fresilli, Mattia Di Segni, Gian Marco Lo Conte, Raponi Flavia, Manuela Garofalo, Renzo Pretagostini, Corrado De Vito, Patrizia Pacini, Vito D’Andrea, Angelo Barbato, Francesco M. Drudi, Marcello Caratozzolo, Vito Cantisani

**Affiliations:** 1 Department of Surgery, Sapienza University of Rome, 00161 Rome, Italy; 2 Department of Radiological, Oncological, and Pathological Sciences, Sapienza University of Rome, Viale Regina Elena 324, 00161 Rome, Italy; 3 Department of Radiology, Ospedale S. Scolastica, Via S. Pasquale, 03043 Cassino, Frosinone Italy; 4 Department of General and Specialty Surgery, Sapienza University of Rome, Viale Regina Elena 324, 00161 Rome, Italy; 5 Department of Public Health and Infectious Diseases, Sapienza University of Rome, 00185 Rome, Italy; 6 ASL Rieti, via del Terminillo 42, Rieti, Italy

**Correction: Journal of Ultrasound (2025) 28:269–280** 10.1007/s40477-025-00989-x

The last author name was incorrectly published as Cantisani Vito instead of Vito Cantisani.

The original article has been corrected.

